# Impact of pathologically confirmed inner lung tumors on nodal upstaging and feasibility of segmentectomy versus lobectomy

**DOI:** 10.1016/j.xjon.2025.101575

**Published:** 2025-12-27

**Authors:** Shinya Tane, Nahoko Shimizu, Naoe Jimbo, Midori Takanashi, Takefumi Doi, Hiroyuki Ogawa, Daisuke Hokka, Yoshitaka Kitamura, Yuki Shimomura, Wataru Nishio, Yoshimasa Maniwa

**Affiliations:** a Division of Thoracic Surgery, Kobe University Graduate School of Medicine, Kobe, Japan; b Department of Chest Surgery, Hyogo Cancer Center, Akashi, Japan; c Clinical and Translational Research Center, Kobe University Hospital, Kobe, Japan; d Department of Diagnostic Pathology, Kobe University Graduate School of Medicine, Kobe, Japan

**Keywords:** inner lesion, lung cancer, prognosis

## Abstract

**Objectives:**

Previous studies reported worse outcomes for radiographically central tumors, but the impact of pathologically confirmed tumor origin remains unclear. This study investigated whether pathologically determined inner lesions are associated with nodal upstaging and poorer prognosis than outer lesions, and examined segmentectomy feasibility versus lobectomy.

**Methods:**

We retrospectively analyzed participants with clinical stage IA (Union for International Cancer Control version 8) non−small cell lung cancer who underwent segmentectomy and lobectomy between November 2007 and December 2022 at 2 Japanese centers. The location of the tumor origin was confirmed pathologically via the Walter classification. Tumors classified as central and intermediate were allocated to the inner group, whereas those classified as peripheral type were allocated to the outer group. The oncologic outcomes were compared between the 2 groups. After propensity score matching analysis on the basis of sex, age, pulmonary function, serum carcinoembryonic antigen level, and radiographic findings, we compared oncologic outcomes in patients who underwent segmentectomy (n = 99) and lobectomy (n = 99) in the inner group.

**Results:**

The cohort comprised inner (n = 654) and outer (n = 1275) groups. Nodal upstaging was greater in the inner group (13.1% [86/654] vs 9.5% [121/1275], *P* = .015). Five-year recurrence-free survival (RFS) was lower in the inner group (73.1%; 95% CI, 69.4%-77.3% vs 79.4%; 95% CI, 76.7%-81.8%, *P* = .002). Multivariable analysis did not identify segmentectomy as significant for RFS (hazard ratio, 0.81; 95% CI, 0.58-1.13; *P* = .20). In matched inner lesions, segmentectomy and lobectomy showed similar RFS (83.6%; 95% CI, 76.3-93.1% vs 76.4%; 95% CI, 66.8-87.4%; *P* = .80).

**Conclusions:**

Although worse prognosis and increased nodal upstaging should be considered in inner primary tumors, segmentectomy is an acceptable treatment option compared with lobectomy for pathologically confirmed inner-located early-stage NSCLC.

**Figure:**
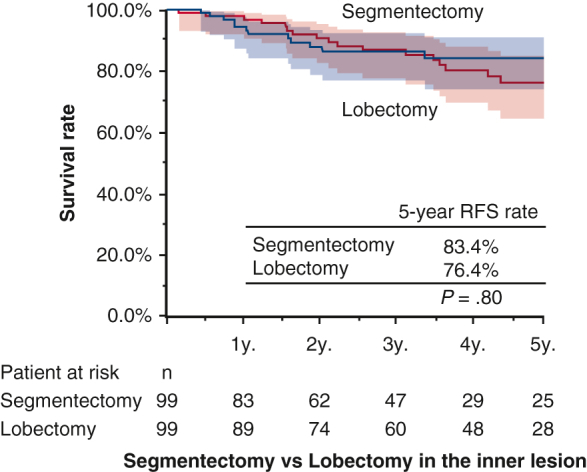
Recurrence-free survival of segmentectomy versus lobectomy in inner lesion with 95% CI.

**Central Message**

Segmentectomy could be an acceptable treatment option compared with lobectomy for pathologically confirmed inner located early stage non−small-cell lung cancer.

**Perspective**

We examined oncologic features of pathologically confirmed inner lesions and the feasibility of segmentectomy for their lesions. Although poor prognosis and nodal upstaging are concerns for inner primary tumors, segmentectomy could be an acceptable treatment option compared with lobectomy.

Recent randomized trials have provided evidence supporting sublobar resection for non−small cell lung cancer (NSCLC) with tumor diameters of 2 cm or less, and segmentectomy has gained popularity among thoracic surgeons.^1^^,^^2^ Moreover, segmentectomy may play an important role as standard therapy for tumors measuring 2 to 3 cm in diameter.^3^, ^4^, ^5^ Thus, the expansion of indications for segmentectomy is anticipated in the near future. However, although all intrapulmonary and hilar lymph nodes within the tumor-bearing lobe can be thoroughly assessed during lobectomy, hilar lymph node dissection may be technically challenging during segmentectomy compared with lobectomy.^6^

Even after preoperative optimal staging, 10% to 20% of the patients with clinical stage I NSCLC are found to have unforeseen lymph node metastasis.^7^^,^^8^ In particular, tumors in the inner locations have a potential risk of unforeseen positive lymph node involvement.^9^, ^10^, ^11^ We have previously demonstrated that segmentectomy for inner small-sized NSCLC yielded equivalent oncologic outcomes in comparison with segmentectomy for outer lesions; however, inner-location tumors have a potential risk of unforeseen positive lymph node involvement during segmentectomy.^10^ To further establish the feasibility of segmentectomy for inner lesions, it is essential to investigate the characteristics of inner lesions more precisely.

The Walter classification is a classical pathologic system that categorizes tumors as “central type,” “intermediate type,” or “peripheral type” on the basis of their origin.^12^ Although several radiographic definitions of tumor centrality exist, previous studies have primarily used radiographic definitions, whereas pathologic classification remains underexplored. Understanding tumor behavior on the basis of pathologic origin provides a biological foundation for risk stratification and may inform future development of improved preoperative imaging criteria and patient selection algorithms.

Therefore, using this definitive classification, we aimed to examine whether pathologically confirmed inner lesions exhibit distinct features and are associated with nodal upstaging and poor prognosis compared with outer lesions. We also aimed to assess the feasibility of segmentectomy versus lobectomy in patients with clinical N0 NSCLC.

## Methods

### Ethical Statement

The Kobe University Hospital institutional review board approved the study (number: B-230220; approved on March 26, 2024), and each participant provided informed consent. Our informed consent statement follows an opt-out approach, so explicit patient consent for the publication of study data was not required.

### Study Design

This was a retrospective, dual-center observational cohort study conducted at Kobe University Hospital and Hyogo Cancer Center between November 2007 and December 2022, in which clinical, radiologic, and pathological data were collected from electronic medical records. The study consisted of 2 major components: first, we compared nodal upstaging and oncologic outcomes between pathologically confirmed inner and outer lesions defined using the Walter pathologic classification; second, we evaluated the oncologic outcomes of segmentectomy versus lobectomy among patients with pathologically confirmed inner lesions.

### Patient Collection

The study reviewed and analyzed the clinicopathologic data and prognosis of patients who underwent segmentectomy or lobectomy for clinical stage I NSCLC between November 2007 and December 2022 at Kobe University hospital and Hyogo cancer center (Union for International Cancer Control, version 8). Preoperative evaluations including chest computed tomography (CT), whole-body ^18^F-fluorodeoxyglucose positron emission tomography–computed tomography, magnetic resonance imaging of the brain, and pulmonary function test were performed to determine clinical stage and treatment strategies. Preoperative tissue diagnosis was determined when feasible through bronchoscopy or CT-guided biopsy. Patients with a history of previous thoracic surgery or lung cancer, those who received induction therapy, were diagnosed with adenocarcinoma in situ or carcinoid tumor, had incomplete resection, underwent hilar nodal dissection only (ND1), or had incomplete clinical information (eg, absence of preoperative positron emission tomography scanning) were excluded. Our basic approach included segmentectomy for tumors ≤3 cm regardless of the consolidation-to-tumor (C/T) ratio, provided adequate surgical margins could be secured, on the basis of our previously reported favorable outcomes.^13^ Selection criteria included intentional segmentectomy for tumors ≤3 cm including both part-solid and pure solid lesions when sufficient margins were achievable, and compromised segmentectomy for patients with poor cardiopulmonary reserve unsuitable for lobectomy.

### Operative Procedure

At the beginning of the study period, the operative procedures, including lobectomy and segmentectomy procedures, were performed by muscle-sparing minithoracotomy (posterolateral or serratus anterior incision) using thoracoscopy as a visual guide. In the subsequent part of the study period, most procedures were performed via the thoracoscopic approach, as described elsewhere.^14^

Both segmentectomy and lobectomy procedures included hilar and mediastinal lymph node dissection (ND2a-1 or ND2a-2). Tumor centrality was not an absolute indication for invasive mediastinal staging at our institutions. This study followed an intention-to-treat analysis. Three patients who were converted from segmentectomy to lobectomy during surgery as the result of insufficient margins (n = 1) or intraoperative nodal upstaging (n = 2) were analyzed in the lobectomy group according to the actual procedure performed.

### Tumor Origin in the Bronchioalveolar Anatomy

All resected specimens were inflated through the corresponding bronchi via gravity drainage with 10% neutral-buffered formalin until fully expanded, then fixed for 1 to 3 days. Routine histopathologic processing with paraffin embedding was performed. Sections (3 μm thick) were stained with hematoxylin and eosin and elastica van Gieson and examined under a light microscope. The Union for International Cancer Control, 8th edition, classification was used for clinical staging. Tumors were staged and graded according to tumor–node–metastasis and World Health Organization classification systems in use at the time of diagnosis (8th edition tumor–node–metastasis and 2015/2021 World Health Organization for recent cases).

**Figure 1:**
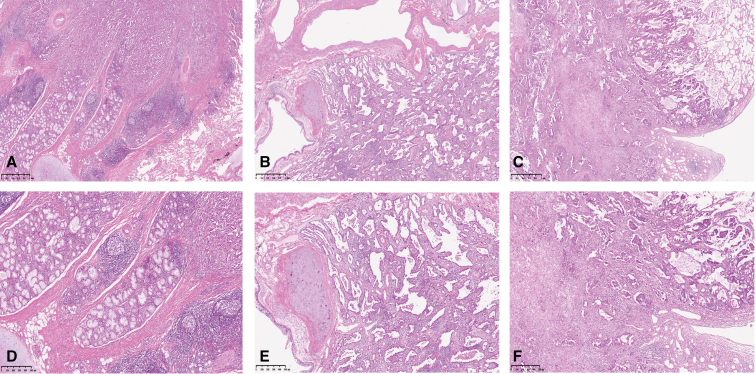
Representative hematoxylin and eosin staining images of central (A [×20] and D [×40]), intermediate (B [×20] and D [×40]), and peripheral adenocarcinoma (C [×20] and F [×40]), according to the Walter classification.

Tumor origin was assessed using the Walter classification, the resected specimens were evaluated by experienced pathologists, and the origin of lung tumors was classified as follows. The representative image of each type is shown in Figure 1:

Central type: Tumors originating from large nominate bronchi, from the main bronchus to the bifurcation of segmental bronchi.

Intermediate type: Tumors arising from smaller bronchi, including all visible branches of the large nominate bronchi.

Peripheral type: Tumors originating from minute bronchi, bronchioles, or alveoli.

### Follow-up

For the first 2 years after the surgical intervention, systemic and local examinations, including blood tests, CT examinations of the chest and abdomen, magnetic resonance imaging of the brain, and bone scintigraphy, were performed at 6-month intervals. Between years 3 and 5, examinations were conducted annually. Long-term follow-up continued indefinitely or for a minimum of 5 years to detect tumor recurrence.

Local recurrence was defined as a recurrent tumor within the same lobe (ie, surgical stump and intrapulmonary metastases). Regional recurrence was defined as involvement of the mediastinal or hilar lymph nodes or a different ipsilateral lobe from the location of the segmentectomy. Distant recurrence was defined as distant metastasis to other organs with diffuse pleural disease.

### Statistical Analysis

Propensity score matching (1:1, caliper width 0.20) was performed for inner lesions using age, sex, tumor size, C/T ratio, carcinoembryonic antigen (CEA) level, maximum standardized uptake value (SUVmax), and pulmonary function. These covariates were selected a priori on the basis of clinical relevance and previous literature. Propensity scores were estimated using logistic regression, and matching was performed without replacement. Balance between matched groups was assessed using standardized mean differences, with a standardized mean difference <0.1 considered acceptable (Table E1). For the matched populations, Wilcoxon signed-rank sum test was used to compare continuous variables and McNemar test was used to compare nominal variables. All analyses followed the intention-to-treat principle, whereby patients were analyzed according to the initially planned surgical procedure, regardless of intraoperative conversions.

Categorical data are presented as number and percentage. Continuous variables are expressed as mean ± standard deviation for normally distributed data or median (interquartile range) for nonnormally distributed data. Normality was assessed visually using histograms. Variables with approximately normal distributions were compared using the Student's *t* test, whereas non-normally distributed variables were compared using the Mann-Whitney *U* test. The χ^2^ test was used for categorical variables.

Recurrence-free survival (RFS) and overall survival (OS) were defined from surgery date to first recurrence/death or the last follow-up. Survival rates were estimated using Kaplan-Meier analysis with log-rank tests for comparison.

Univariable and multivariable Cox regression analyses were performed to identify factors associated with RFS. Variables were selected on the basis of established clinical relevance and previous literature^10^ and included age (>74 years), sex, pulmonary function, C/T ratio, tumor size, histology, CEA level, SUVmax, and surgical procedure. Statistical significance was set at *P* < .05 for 2-tailed tests, and analyses were performed using JMP 14 software (SAS Institute Inc).

## Results

### Patient Enrollment

During the study period, 16 patients with incomplete resection (2 segmentectomies and 14 lobectomies) were excluded from the analysis. All of these cases had residual disease including malignant pleural effusion, pleural dissemination, or chest-wall invasion. A total of 1929 patients with clinical stage IA NSCLC underwent lobectomy or segmentectomy with hilar and mediastinal nodal dissection (ND2a-1 or ND2a-2). This study cohort comprised 42, 612, and 1275 patients with the central, intermediate, and peripheral type tumors, respectively. As we defined the central type and the intermediate type as inner lesion, and the peripheral type as the outer lesion, our cohort was divided into the inner group (n = 654) and the outer group (n = 1275).

### Patient Characteristics in the Inner and Outer Lesions

The characteristics of patients in the inner and outer groups are summarized in Table 1. Patients with inner lesions were significantly older (*P* = .043), had lower pulmonary function (forced expiratory volume in 1 second [FEV1.0]; *P* = .002, FEV1.0/forced vital capacity [FVC]; *P* = .005), a greater frequency of squamous cell carcinoma (*P* = .014), larger tumor diameters (*P* < .001), and greater SUVmax values (*P* < .001).

**Table 1:** Clinicopathologic data (N = 1929) in the entire cohort

Variable	Inner (n = 654)	Outer (n = 1275)	*P* value
Gender			.043
Male	359 (55)	761 (60)	
Female	295 (45)	514 (40)	
Age, y	70.2 ± 9.3	68.8 ± 9.0	<.001
BMI, kg/m^2^	22.5 ± 3.2	22.8 ± 3.7	.036
Smoking			.028
Yes	351 (53.7)	751 (58.9)	
No	303 (46.3)	524 (41.1)	
FEV1.0, L	2.18 ± 0.57	2.28 ± 0.61	.002
FEV1.0/FVC, %	72.3 ± 9.6	73.6 ± 9.9	.005
CEA (>5), ng/dL	157 (24.1)	281 (22.6)	.30
Histopathologic type			.40
Adenocarcinoma	527 (80.6)	1059 (83.0)	
Squamous cell carcinoma	95 (14.4)	164 (12.9)	
Others	32 (4.9)	52 (4.1)	
Tumor diameter on CT	22 ± 8	20 ± 8	<.001
C/T ratio			.003
≤0.5	155 (24)	389 (27)	
0.5-1	171 (26)	333 (30)	
1	328 (50)	553 (43)	
SUV max	4.26 ± 4.11	3.82 ± 3.80	.014
Operative procedure			
Lobectomy	553	1062	
Segmentectomy	101	213	
pN+	86 (13.1)	121 (9.5)	.015
Lymphatic invasion	150 (22.9)	273 (21.4)	.45
Vascular invasion	185 (28.3)	315 (24.7)	.094
Adjuvant therapy	187 (28.5)	372 (29.1)	.78

Data are expressed as the mean ± standard deviation, median [interquartile range], or n (%). *BMI*, Body mass index; *FEV1.0*, forced expiratory volume in 1 second; *FVC*, forced vital capacity; *CEA*, carcinoembryonic antigen; *CT*, computed tomography; *C/T*, consolidation-to-tumor; *SUV*, standardized uptake value.

Regarding nodal upstaging, inner lesions showed a significantly greater incidence of lymph node metastasis compared with outer lesions (13.1% vs 9.5%, *P* = .015). When limited to adenocarcinoma cases, nodal upstaging followed the same trend as the overall cohort. However, in squamous cell carcinoma, lymph node upstaging did not differ significantly by tumor location (6.3% vs 10.3%, *P* = .26).

### Comparison of Prognosis Associated With Inner and Outer Lesions

**Figure 2:**
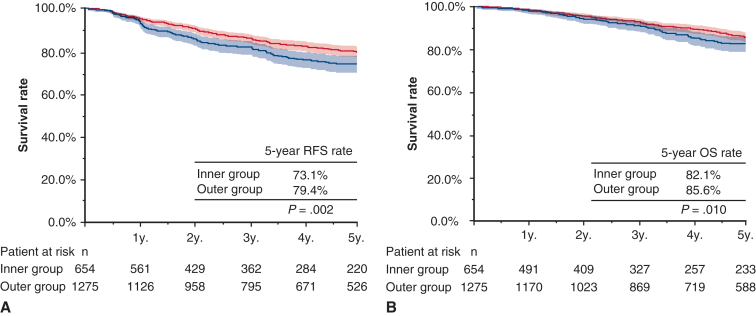
RFS and OS (A and B) in patients with clinical stage IA according to tumor location. *Shading* indicates 95% CI. *RFS*, Recurrence-free survival; *OS*, overall survival.

The median follow-up period was 60.7 months (interquartile range, 31.0-89.7 months). As shown in Figure 2, the 5-year RFS was 73.1% (95% CI, 69.8%-77.7%) in the inner group and 79.4% (95% CI, 76.9%-82.0%) in the outer group (*P* = .002), indicating significantly lower RFS in inner lesions. The 5-year OS was 82.1% (95% CI, 78.6%-85.8%) in the inner group and 85.6% (95% CI, 83.3%-87.9%) in the outer group (*P* = .010), also showing that the inner lesions were associated with significantly lower OS than outer lesions.

**Figure E1:**
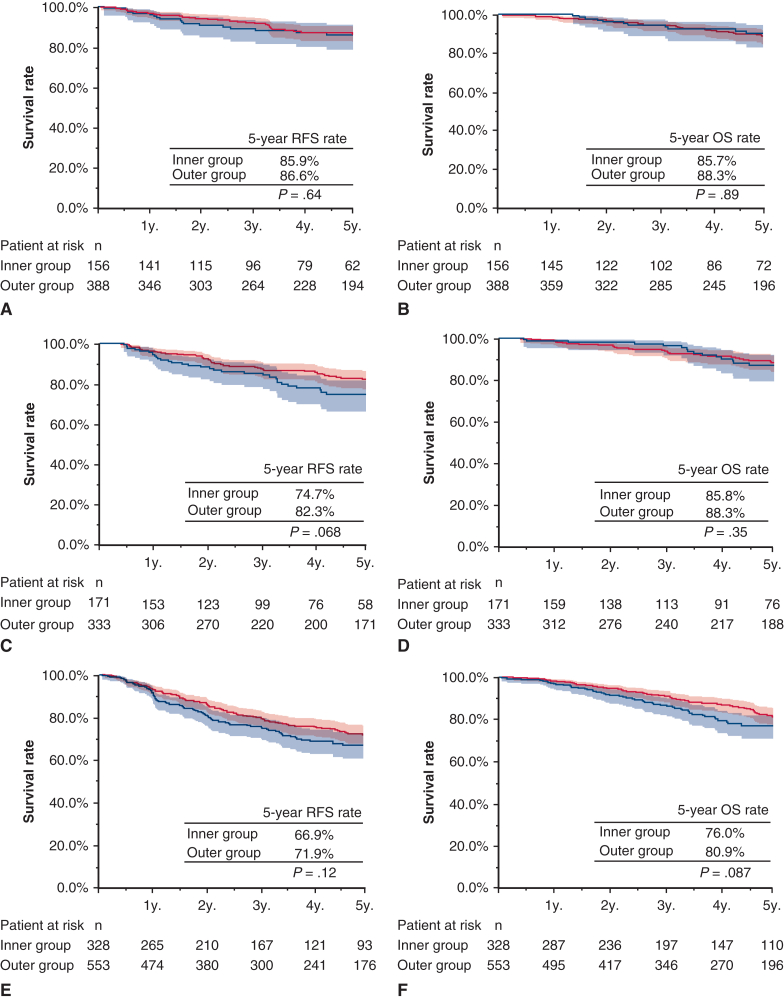
RFS and OS (A and B) according to C/T ratio <0.5 (A and B), 0.5-1 (C and D), and >1 (E and F). *Shading* indicates 95% CI. *RFS*, Recurrence-free survival; *OS*, overall survival; *C/T*, consolidation-to-tumor.

According to the stratified analysis by the C/T ratio, RFS showed no significant differences between the inner and outer lesion in the tumor with C/T < 0.5 (85.9%; 95% CI, 82.3-90.0%) versus 86.6% (95% CI, 78.6-91.0%: *P* = .64), whereas inner lesion showed worse outcomes in the tumor with C/T = 0.5-1 (74.7%; 95% CI, 66.2-81.7% vs 82.3%; 95% CI, 77.3-86.4%; *P* = .068), and C/T = 1 (66.9%; 95% CI, 60.7-72.7% vs 71.9%; 95% CI, 67.2-76.2%: *P* = .12). As is the similar with RFS, OS showed no significant differences between inner and outer lesion in the tumor with C/T = <0.5 (85.7%; 95% CI, 84.5%-91.8% vs 88.3%; 95% CI, 77.9-94.5%: *P* = .89), and C/T = 0.5-1 (85.8%; 95% CI, 77.9%-91.2% vs 88.3%; 95% CI, 83.8%-91.7%; *P* = .35), whereas inner lesion showed worse outcomes in the tumor with C/T = 1 (76.0%; 95% CI, 69.9%-81.2% vs 80.9%; 95% CI, 76.4%-84.8%: *P* = .087) (Figure E1).

When analysis was limited to patients with pN0, inner lesions still showed worse 5-year RFS than outer lesions (79.9%; 95% CI, 75.8-83.6% vs 83.3%, 95% CI, 81.2%-81.6%: *P* = .003), whereas the OS was 86.5%; 95% CI, 82.7-89.6% versus 87.2%; 95% CI, 84.9%-89.5% *P* = .097. According to the stratified analysis by the C/T ratio, RFS and OS showed the same trend as the entire cohort.

**Figure 3:**
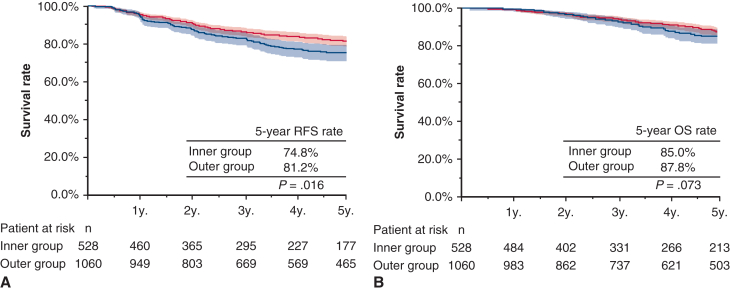
RFS and OS (A and B) in patients with adenocarcinoma according to tumor location. *Shading* indicates 95% CI. *RFS*, Recurrence-free survival; *OS*, overall survival.

**Figure 4:**
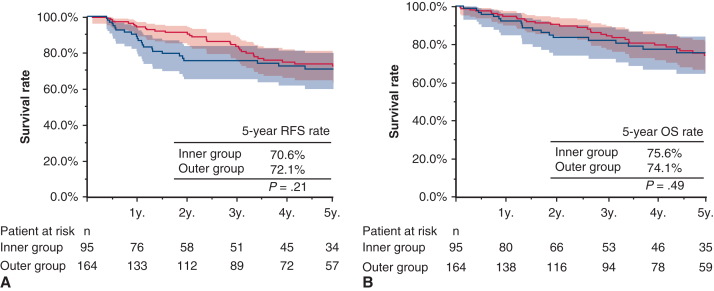
RFS and OS (A and B) in patients with squamous cell carcinoma according to tumor location. *Shading* indicates 95% CI. *RFS*, Recurrence-free survival; *OS*, overall survival.

Next, RFS and OS were analyzed according to histologic subtype. For adenocarcinoma, the 5-year RFS was 74.8% (95% CI, 71.0%-79.7%) in the inner group and 81.2% (95% CI, 78.7%-84.0%) in the outer group (*P* = .016). The 5-year OS was 85.0% (95% CI, 81.3%-88.9%) in the inner group and 87.8% (95% CI, 85.5%-92.2%) in the outer group (*P* = .073), suggesting a poorer prognosis with inner lesions than with the outer lesions (Figure 3). In contrast, as shown in Figure 4, when limited to the cases with squamous cell carcinoma, no significant difference was noted between the 2 groups regarding RFS and OS (5-year RFS, 70.6%; 95% CI, 62.0%-82.3% vs 72.1%; 95% CI, 64.3%-81.0%; *P* = .21; 5-year OS, 75.6%; 95% CI, 66.5%-86.1% vs 74.1%; 95% CI, 66.5%-82.8%, *P* = .49).

### Univariate and Multivariate Analyses for RFS in the Entire Cohort (n = 1929)

To identify which clinical factors including surgical procedure could be independent prognostic factors, we evaluated univariable and multivariable analyses in the entire cohort of 1929 patients (Table 2). Age cutoff of 74 years was based on median age of the cohort. SUVmax cutoff of 3.2 was determined using receiver operating characteristic curve analysis for optimal discrimination of recurrence risk. Multivariable analysis identified age >74 years (hazard ratio [HR], 1.39; 95% CI, 1.14-1.70; *P* = .001), pure solid tumor (HR, 1.56; 95% CI, 1.26-1.92; *P* = .007), tumor diameter >20 mm (HR, 1.24; 95% CI, 1.01-1.54; *P* = .040); CEA >5 (HR, 1.78; 95% CI, 1.45-2.17; *P* < .001), and SUVmax >3.2 (HR, 2.24; 95% CI, 1.95-3.01; *P* < .001) as independent predictors of recurrence (Table E2). Neither segmentectomy (HR, 0.81; 95% CI, 0.58-1.13, *P* = .20) nor inner location (HR, 1.15; 95% CI, 0.94-1.40, *P* = .15) retained significance in the multivariable model.

**Table 2:** Univariable and multivariable analysis for recurrence-free survival in the all patients (n = 1929)

Variable	Univariate			Multivariate		
	HR	95% CI	*P* value	HR	95% CI	*P* value
Age (74)	1.76	1.41-2.19	<.001	1.39	1.14-1.70	.001
Sex (male)	1.65	1.35-2.02	<.001	1.20	0.96-1.50	.094
EFV1.0/FVC (<0.7)	1.49	1.22-1.82	<.001	1.01	0.87-1.33	.47
Pure solid	2.24	1.80-2.79	<.001	1.56	1.26-1.92	.007
Tumor diameter (>20 mm)	1.53	1.25-1.89	.012	1.24	1.01-1.54	.040
Histology (adenocarcinoma)	0.52	0.41-0.66	<.001	0.94	0.75-1.19	.62
CEA (>5)	2.22	1.82-2.69	<.001	1.78	1.45-2.17	<.001
SUVmax (3.2)	3.25	2.65-4.00	<.001	2.24	1.45-2.17	<.001
Segmentectomy (vs lobectomy)	0.63	0.45-0.86	.003	0.81	0.58-1.13	.20
Inner location (vs outer lesion)	1.47	1.11-1.63	.002	1.15	0.94-1.40	.15

*HR*, Hazard ratio; *FEV1.0*, forced expiratory volume in 1 second; *FVC*, forced vital capacity; *CEA*, carcinoembryonic antigen; *SUVmax*, maximum standardized uptake value.

### Comparison of Oncologic Prognosis in Patients With Inner Lesions Who Underwent Either Segmentectomy or Lobectomy

**Figure 5:**
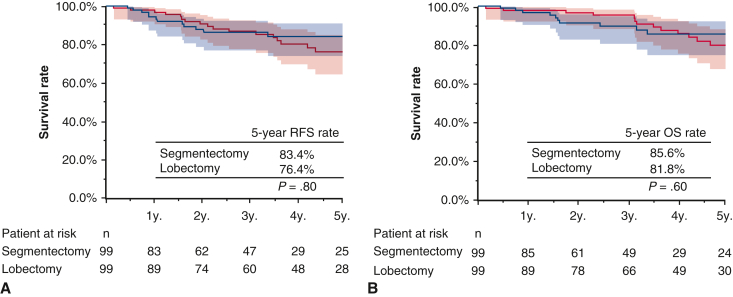
RFS and OS (A and B) in matched patients with the inner lesion who underwent segmentectomy (n = 99) and lobectomy (n = 99). *Shading* indicates 95% CI. *RFS*, Recurrence-free survival; *OS*, overall survival.

Among 654 patients with inner lesions (segmentectomy n = 1017, lobectomy n = 553), propensity score matching identified 99 patients each for analysis (Tables E3). The mean follow-up period was 50 ± 34 months. Five-year RFS rates were 83.6% (95% CI, 76.3%-93.1%) and 76.4% (95% CI, 66.8%-87.4%) for segmentectomy and lobectomy, respectively (*P* = .80). Five-year OS rates were 85.6% (95% CI, 66.8%-87.4%) and 81.8% (95% CI, 70.3%-90.7%) for segmentectomy and lobectectomy, respectively (*P* = .60) (Figure 5, *A* and *B*).

The recurrence patterns of the matched patients are shown in Table E3. During the follow-up assessments, 39 of the 198 patients showed disease recurrence. No local recurrence was observed in either group. Regional recurrence was noted in 9 patients (the residual lobe in 4 patient and the hilar and mediastinal lymph nodes in 5 patients) in the segmentectomy group and 10 patients (the residual lobe in 5 patients, the hilar and mediastinal lymph nodes in 4 patients, and pleural dissemination in 1 patient) in the lobectomy group. Distant metastasis or combined distant and local recurrence was observed in 2 patients in the segmentectomy group and 6 patients in the lobectomy group.

## Discussion

Many studies have identified risk factors for unexpected postoperative lymph node metastasis in patients with cN0 NSCLC, with several reports demonstrating that central tumor location is associated with greater incidence of lymph node metastasis. Decaluwe and colleagues^9^ reported that central tumors in the inner one third of the lung had 4 times greater odds of unexpected nodal upstaging. Kawamoto and colleagues^11^ found that inner two thirds tumors had greater occult hilar lymph node metastasis rates (21.5% vs 7.4%). However, these studies used varying radiographic definitions of tumor centrality, which are subjective and inconsistent across studies. The present study provided objective, biologically relevant categorization. Although treatment decisions rely on preoperative imaging, understanding tumor behavior based on pathologic origin provides biological validation of current risk assessment approaches and confirms that inner lesions are associated with greater lymph node metastasis rates.

Recently, multi-institutional randomized clinical trials (JCOG0802/WJOG4607L, JCOG1211, and CALGB140503) demonstrated the clinical value of segmentectomy in NSCLC. However, this benefit is limited to patients with small peripheral NSCLC. In our previous report, we demonstrated that segmentectomy for inner small-sized NSCLC, defined by a novel 3-dimensional measurement method, showed favorable oncologic outcomes comparable to segmentectomy for outer lesions.^10^ Tumor invasiveness, rather than tumor centrality, was identified as the most important prognostic factor for RFS after segmentectomy. However, lymph node metastasis was more frequent in inner lesions compared with outer lesions. Regarding the comparison between segmentectomy and lobectomy, several reports have demonstrated that segmentectomy had a comparable impact on prognosis to lobectomy in inner lesions.^15^^,^^16^ In order to expand the indication for segmentectomy to central lesions, a more detailed understanding of the clinical and pathologic characteristics of inner lesions is essential.

One of the concerns during segmentectomy is insufficient hilar lymph node dissection. The JCOG0802 trial revealed that the recurrence rate with ipsilateral lymph node metastasis was not significantly different between segmentectomy and lobectomy.^1^ In addition, our previous study demonstrated that segmentectomy for inner-located small-sized non−small cell lung tumors could be an acceptable treatment compared with lobectomy.^15^ Nonetheless, attention must be given to lymph node metastasis, particularly in cases of innerlocated adenocarcinoma during segmentectomy.

An important finding of this study is that oncologic outcomes in early-stage NSCLC are primarily determined by indicators of biological aggressiveness rather than the extent of surgical resection. Our multivariable analyses demonstrated that biological markers—including SUVmax >3.2, tumor diameter >20 mm, pure solid tumor morphology, and elevated CEA levels—were independent predictors of recurrence, whereas the choice between segmentectomy and lobectomy did not significantly impact oncologic outcomes. This finding was consistent across both the entire cohort (n = 1929) and the propensity-matched inner lesion cohort (n = 198), suggesting that tumor biology, rather than surgical approach, is the primary determinant of prognosis in patients with localized lung cancer.

In the current study, although adenocarcinomas in central tumors exhibited more frequent occult lymph node metastasis compared with peripheral tumors, there was no significant difference in lymph node metastasis between central and peripheral tumors in squamous cell carcinoma. This discrepancy may be related to different biological characteristics between histologic subtypes. Although not statistically significant, epidermal growth factor receptor positivity showed a trend toward greater frequency in inner lesions (46.7% vs 39.2%, *P* = .18), suggesting potential biological differences that warrant further investigation. Although not significantly different, these genetic changes may impact the occurrence of occult lymph node metastasis in adenocarcinoma. In contrast, in squamous cell carcinoma, the squamous injury response induced by smoking may drive carcinogenesis through the activation of SOX-2 and phosphatidylinositol 3-kinase pathways, rather than tumor location.^17^ Further research on the genetic differences related to tumor origin would be valuable for future studies.

This study has several limitations. First, it was a retrospective, 2-center study, which introduces biases such as patient selection and measurement bias. Second, a longitudinal mixed surgical approach (thoracotomy or thoracoscopic) may influence the precision of lymph node dissection.

Third, we did not provide details on the number of lymph nodes, specific lymph node stations examined, or breakdown of stations sampled in each surgical group, although lymph node counts are highly variable and unreliable because of fragmentation.^18^ Fourth, the inherent differences in tumor biology and patient characteristics between lobectomy and segmentectomy groups represent a major limitation. Although propensity score matching was performed, residual selection bias may persist. Lastly, systematic collection of detailed histopathologic features including tumor spread thought air space, resection margin measurements, and types of resected segments was not recorded.

In conclusion, nodal upstaging and poor prognosis should be considered in the case of inner primary tumors but not in squamous cell carcinoma. Although worse prognosis and increased nodal upstaging should be considered in inner primary tumors, segmentectomy is an acceptable treatment option compared with lobectomy for pathologically confirmed inner-located early-stage NSCLC when patients are appropriately selected on the basis of biological markers.

## Conflict of Interest Statement

The authors reported no conflicts of interest.

The *Journal* policy requires editors and reviewers to disclose conflicts of interest and to decline handling or reviewing manuscripts for which they may have a conflict of interest. The editors and reviewers of this article have no conflicts of interest.

## Figures and Tables

**Table E1:** SMDs before and after propensity-score matching

Variable	Prematched SMD	Postmatched SMD
Gender (male/female)	0.114	0.091
Age, y, mean ± SD	0.269	0.097
Tumor diameter, mm	0.758	0.083
FEV1.0, L, mean ± SD	0.220	0.034
FEV1.0/FVC, %, L, mean ± SD	0.046	0.094
C/T ratio	0.105	0.059
CEA (>5), ng/dL	0.015	0.092
SUVmax	0.442	0.015

*SMD*, Standardized mean difference; *SD*, standard deviation; *FEV1.0*, forced expiratory volume in 1 second; *FVC*, forced vital capacity; *C/T*, consolidation-to-tumor; *CEA*, carcinoembryonic antigen; *SUVmax*, maximum standardized uptake value.

**Table E2:** Clinicopathologic data of the patients who underwent segmentectomy and lobectomy in the inner group (N = 654)

Variable	Segmentectomy (n = 101)	Lobectomy (n = 553)	*P* value
Sex			.33
Male	51	308	
Female	50	245	
Age, y	72.4 ± 8.9	69.8 ± 9.3	.014
BMI, kg/m^2^	22.4 ± 3.5	22.4 ± 3.1	.98
Smoking			.61
Yes	57	294	
No	44	259	
FEV1.0, L	2.06 ± 0.69	2.20 ± 0.57	.030
FEV1.0/FVC, %, L	73.4 ± 9.6	73.9 ± 10.9	.46
CEA (>5), ng/dL	25 (24.7)	132 (28.3)	.92
Histopathologic type			.84
Adenocarcinoma	82 (81.2)	446 (80.7)	
Squamous cell carcinoma	15 (14.9)	79 (14.3)	
Others	4 (4.0)	28 (5.1)	
Tumor diameter on CT	18 ± 6	23 ± 6	<.001
C/T ratio			.008
≤0.5 (%)	22 (21.8)	119 (21.5)	
0.5-1 (%)	36 (35.6)	149 (26.9)	
1 (%)	43 (42.6)	285 (51.5)	
SUV max	2.78 ± 2.28	4.53 ± 4.12	<.001
pN+ (%)	6 (5.9)	80 (14.4)	.018
Vascular invasion, %	16 (15.8)	169 (31.7)	.002
Lymphatic invasion, %	9 (8.9)	141 (25.5)	<.001
Adjuvant therapy, %	7 (6.9)	180 (32.5)	<.001
Recurrence pattern, %			.583
Local	0	2 (0.3)	
Regional	9 (8.9)	65 (11.8)	
Distant	6 (5.9)	48 (8.7)	
Regional + distant	2 (2.0)	42 (7.6)	

Data are expressed as the mean ± standard deviation, median [interquartile range], or n (%). *BMI*, Body mass index; *FEV1.0*, forced expiratory volume in 1 second; *FVC*, forced vital capacity; *CT*, computed tomography; *C/T*, consolidation tumor; *CEA*, carcinoembryonic antigen; *SUVmax*, maximum standardized uptake value.

**Table E3:** Clinicopathologic data of the matched patients who underwent segmentectomy and lobectomy in the inner group (N = 198)

Variable	Segmentectomy (n = 99)	Lobectomy (n = 99)	*P* value
Gender	50/49	45/54	.47
Male	50	45	
Female	49	54	
Age, y	72.4 ± 8.9	73.6 ± 7.3	.30
BMI, kg/m^2^	22.5 ± 3.2	22.2 ± 3.1	.55
Smoking			.32
Yes	56	49	
No	43	50	
FEV1.0, L	2.06 ± 0.69	2.04 ± 0.50	.54
FEV1.0/FVC, %, L	73.4 ± 9.6	72.3 ± 9.5	.46
CEA (>5), ng/dL	24 (24.2)	28 (28.3)	.51
Histopathologic type			.40
Adenocarcinoma	80 (80.8)	88 (88.9)	
Squamous cell carcinoma	15 (15.2)	8 (8.1)	
Others	4 (4.0)	3 (3.0)	
Tumor diameter on CT	19 ± 6	19 ± 6	.56
C/T ratio			.46
≤0.5	22 (22.2)	26 (26.2)	
0.5-1	35 (35.3)	27 (27.3)	
1	42 (42.4)	46 (46.5)	
SUVmax	2.65 ± 2.51	2.70 ± 2.65	.91
pN+	6 (6.0)	8 (8.0)	.57
Lymphatic invasion	15 (15.2)	16 (16.1)	.82
Vascular invasion	9 (9.1)	17 (17.2)	.092
Adjuvant therapy	7 (7.1)	19 (19.2)	.010
Recurrence pattern			.48
Local	0	0	
Regional	9 (9.1)	10 (10.1)	
Distant	6 (6.0)	6 (6.1)	
Regional + distant	2 (2.0)	6 (6.1)	

Data are expressed as the mean ± standard deviation, median [interquartile range], or n (%). *BMI*, Body mass index; *FEV1.0*, forced expiratory volume in 1 second; *FVC*, forced vital capacity; *CT*, computed tomography; *C/T*, consolidation/tumor; *CEA*, carcinoembryonic antigen; *SUVmax*, maximum standardized uptake value.
